# Sexual experiences and behaviours in the offspring generation of the Avon Longitudinal Study of Parents and Childhood

**DOI:** 10.12688/wellcomeopenres.21687.1

**Published:** 2024-05-09

**Authors:** Yasmin Iles-Caven, Jean Golding

**Affiliations:** 1 Population Health Sciences, Bristol Medical School, University of Bristol, Bristol, England, BS8 2BN, UK

**Keywords:** ALSPAC, sexual experiences, sexual behaviours, childhood, adolescence, young adults, sexual functioning, risky behaviour

## Abstract

Previous research on child/teenage sexual experiences has largely focussed on negative outcomes such as teen pregnancy or acquiring sexually transmitted infections and are mainly cross-sectional. Longitudinal research is required to assess normal sexual development and the attainment of psychologically healthy attitudes towards sexuality. The Avon Longitudinal Study of Parents and Children (ALSPAC) has administered questions on relationships and sexual experiences from the age of 11 years to the index children. This data note describes these data.

## Background

More than 25% of adolescents in the UK report sexual activity before age 16 ( Jessor, 1998) with mean age of sexual debut (coitarche or age at first intercourse) at 14 years for both boys and girls ( Brooks-Gunn & Furstenberg, 1989). Adolescents are at higher risk of various negative health consequences associated with early coitarche and unsafe sexual activity, including sexually transmitted diseases (STIs) and unintended pregnancy (see Kotchick *et al.*, 2001 for a review; Mårdh *et al.*, 2000). Results from the UK’s NATSAL-3 report that over 45% of pregnancies occurring between ages 16–19 were unplanned and that coitarche <16 years was one strongly associated risk factor (aged adjusted OR 2.85) ( Wellings *et al.*, 2013); as well as ‘sexual competence’ at first sexual intercourse (defined as fulfilling four criteria: contraceptive protection; autonomy of decision (not due to external influences); that both partners were “equally willing”; and that it happened at the “right time” ( Palmer *et al.*, 2017). In addition, romantic and sexual relationships that begin at young ages are associated with an increased risk of maladaptive outcomes such as lower educational and occupational attainment; other risky behaviour such as alcohol and drug use (e.g. Ary *et al.*, 1999; Fergusson *et al.*, 1994; James *et al.*, 2012; and Sprecher *et al.*, 2019 for a review); and negative influences on mental health, relationship skills and sexual functioning in later life (e.g. Kahn & Halpern, 2018; Sandfort *et al.*, 2008)).

Although previous research has examined involvement in coital sexual activity in children from the age of nine upwards, such studies are mainly cross-sectional and focus on only one or two associated factors and single outcomes (e.g. involvement with a sexually transmitted infection (STI) or teen pregnancy). Exceptions include the National Longitudinal Study of Adolescent Health in the United States which has found higher levels of STIs in adolescents with early coitarche and more sexual partners (e.g. Kaestler *et al.*, 2005). More longitudinal research is required to develop multi-system models which include individual, familial and social factors, and which facilitate the examination of interactions between all of these factors on not only the problems of STIs and teenage pregnancy but also on normal sexual development and the attainment of psychologically healthy attitudes towards sexuality.

To this end, the Avon Longitudinal Study of Parents and Children (ALSPAC) administered questions on sexual experiences known as ‘Romantic relationships’ from the age of 11.5 years to the index offspring and these are described in this data note. In addition, we describe data concerning sexual experiences collected when they were 21 and 23 years of age.

This paper does not cover adverse childhood events (ACEs) to the index children, nor other data on sexual abuse, which are detailed/analysed elsewhere (e.g. Herbert *et al.*, 2021; Houtepen *et al.*, 2018; Lawn *et al.*, 2018; Lussier *et al.*, 2023; Mars *et al.*, 2014; Sidebotham & ALSPAC Study team, 2000; Sidebotham *et al.*, 2001; Sidebotham *et al.*, 2002; Troy *et al.*, 2021).

A companion data note details early sexual experiences in the mothers (and their partners) (G0s) of these offspring (G1s), ( Iles-Caven & Golding, 2024). 

## Methods

### The Avon Longitudinal Study of Parents and Children (ALSPAC)

All pregnant women (G0 cohort) resident in the Avon area of south-west England with expected dates of delivery between April 1991 and December 1992 inclusive were invited to enrol in ALSPAC. The initial number of pregnancies enrolled was 14,541, resulting in 13,988 infants who were alive at age 12 months. Additional phases of enrolment are described in more detail in the cohort profile papers ( Boyd *et al.*, 2013; Fraser *et al.*, 2013; Northstone *et al.*, 2019). These resulted in a total sample size for analyses using any data collected after the age of seven of 15,447 pregnancies resulting in 15,658 fetuses; of these, 14,901 children (G1 cohort) were alive at 1 year of age. Since 2014, study data have been collected and managed using REDCap electronic data capture tools hosted at the University of Bristol. REDCap (Research Electronic Data Capture) is a secure, web-based software platform designed to support data capture for research studies ( Harris *et al.*, 2009). For those participants who prefer to complete paper versions these are also offered. Completed paper questionnaires are scanned into electronic data using Teleform data capture software. Please note that the study website contains details of all the data that is available through a fully searchable data dictionary and variable search tool: http://www.bristol.ac.uk/alspac/researchers/our-data/.

Annual hands-on assessment clinics were held for all the offspring from the age of 7 years and this paper uses data collected at Focus@11 (F@11 at 11.5yrs+); TeenFocus1 (TF1 at 12.5yrs+) and the Fast Track version thereof; TeenFocus2 (TF2 at 13.5yrs+); TeenFocu3 (TF3 at 14.5yrs+) and TeenFocus 4 (TF4 at 17 years of age). Additionally, we report on data collected via self-completion questionnaires administered when the young people were 21+ and 23+ years of age.

Ethical approval for the ALSPAC study was obtained from the ALSPAC Ethics and Law Committee (ALEC; IRB00003312) and the Local Research Ethics Committees. Detailed information on the ways in which confidentiality of the cohort is maintained may be found on the study website: http://www.bristol.ac.uk/alspac/researchers/research-ethics/. All methods were performed in accordance with the relevant guidelines and regulations at the time. Informed consent for the use of data collected via questionnaires was assumed from participants following the recommendations of the ALSPAC Ethics and Law Committee at the time ( Birmingham, 2018). Signed consent was obtained for information obtained in the hands-on clinics.

## Description of data collected

Basic demographics of the offspring (G1) cohort are presented in Table 1, where we also have specified those demographics related to their upbringing (i.e. maternal Index of Multiple Deprivation, living in an urban/rural environment, and maternal social class and educational attainment). 

**Table 1.** Basic demographics of the young people (YP) including the environment in which they were brought up.

Sociodemographic G1 (variable name)	N	%
**First born**		
YP was the eldest child (kd431)	4902	44.4
YP was a member of a twin pregancy (kb610)	264	2.3
**Ethnicity of YP** (c804)		
White	11394	94.9
Non-white	604	5.1
**Highest Education level of YP (at age 26+)** (YPF7970)		
GCSE or equivalent	475	13.5
A level & higher NVQs	1393	38.8
Degree or higher	1713	47.9
**YP’s current relationship status (at age 29+)** (YPJ2500)		
Married	975	22.8
In civil partnership	105	2.5
Engaged	597	14.0
Other partner type (excl. any of above)	1631	38.2
No partner	963	22.5
**YP lives with partner (at age 29+)** (YPJ2510)		
Yes, all the time	2786	84.3
Yes, some of the time	208	6.3
No	312	9.4
**YP is currently a parent (age 29+)** (YPJ6000)		
Yes	1132	27.0
No	3062	73.0
***YP raised in the following:***		
**Maternal social class** (c755)		
Non-manual (I-IIINM)	7990	80.1
Manual (IIIM-V	1983	19.9
**Maternal housing tenure in pregnancy** (a006)		
Owner occupied	9732	73.1
Rented/all other	3577	26.9
**Maternal Urban/Rural location** (Jan1993ur01ind_m)		
Urban	11676	90.2
Town & fringe	528	4.1
Village	531	4.1
Hamlet/isolated dwelling	202	1.6
**Maternal IMD:** (Jan2014md2010q5)		
1 least deprived	3936	30.5
2	2939	22.8
3	2281	17.7
4	2108	16.3
5 most deprived	1631	12.6

Table 2 indicates the numbers of respondents at each time point of data collection in the Focus clinics, and questionnaires at 21 and 23 years for the males and females separately. The majority of frequencies described in this paper have not been split by sex assigned at birth to protect confidentiality of participants, where numbers are small. Note that from the age of 15.5 years more females than males continued to participate.

**Table 2.** Assigned sex at birth (variable kz021) by attendance at clinics/questionnaires completed. Please note that not every clinic attendee completed every session, nor that respondents completed every section or question within a questionnaire.

Sex at birth	F@11 11.5yrs	TF1 12+yrs	TF2 13+yrs	TF3 15+yrs	TF4 17+yrs	21yrs	23yrs
*kz021*	N (%)	N (%)	N (%)	N (%)	N (%)	N (%)	N (%)
Male	3518 (49.1)	3356 (49.1)	3018 (49.1)	2600 (47.4)	2277 (43.6)	1231 (35.5)	1479 (35.0)
Female	3641 (50.9)	3482 (50.9)	3129 (50.9)	2915 (52.6)	2940 (56.4)	2232 (64.5)	2743 (65.0)
**Total**	**7159**	**6838**	**6147**	**5515**	**5217**	**3463**	**4222**

### Romantic Relationships computerised interview at Focus at 11, TF1-TF3

From the age of 11 years, the G1 cohort had answered computerised sets of questions known as ‘Romantic Relations’ during the hands-on assessment clinics.

The questionnaire was derived from Hansen *et al.*’s (1999) Adolescent Sexuality Activity Index (ASAI) and incorporated into Focus@11 because it was deemed important, as the offspring entered their second decade, to gain an understanding of how they managed the transition from childhood to sexually active adolescents and eventually adults. The ASAI is a 13-item measure asking about progressive pre-coital sexual behaviours designed for use with adolescents, which was slightly modified for the F@11 sweep. The interview was performed on a computer and the tester assured the child of the confidentiality of their answers and how the interview worked. If the child answered No or did not respond to the question regarding spending time alone with the opposite sex, the interview was terminated; therefore, only the 1548 children who answered Yes were presented with questions on kissing, and so on. For each activity the child was asked whether they had done this in the previous year, and if so, whether they had enjoyed it.

All questions specified the experiences as being with another young person. The first four clinics comprised questions on friendships; handholding, cuddling, kissing, touching (under clothes), being undressed, fondling of genitals, oral sex (not at F@11), and sexual intercourse. Most of these activities had follow-on questions, for example, on enjoyed the experience; and for TF1 onwards: wanting the experience, was coerced, had drunk alcohol (and level of intoxication) or taken drugs prior to the event, reasons for doing so, regret, etc. At older time points, the respondents were also asked about condom availability and use along with other specified forms of contraception (See Extended Data Supplementary Table 1).

At all time points, questions elicit details about the sex of the young person’s friends and/or whether any of the sexual experiences involved the opposite sex (and from TF2 same-sex partners). Later questions (from TF3) ask the young person to explicitly identify their own gender.

A much smaller set of questions were administered at TF4 (17yrs) for a sub-study on chlamydia (funded by NHS Bristol) on sexual activities and tested urine for chlamydia. For more information on the results of the chlamydia project see Crichton *et al.* (2014). Full lists of all the questions asked at the clinics, along with the variable names are listed in Extended Data Supplementary Table 1 (F@11, TF1-4). 

At the ages of 21+ and 23+ years a slightly different set of questions was asked via self-completion questionnaires, which could be completed online or as paper versions as per the respondent’s preference (see Extended Data, Supplementary Table 2 for the complete list and variable names).

## Observations

Table 3 gives percentage frequencies (of clinic attendees) for selected physical experiences only with members of the opposite sex to allow comparison between F@11, TF1, TF2 and TF3 since at F@11 and TF1 the question specified the opposite sex, whereas TF2 and TF3 asked whether the experience occurred with members of the same sex or opposite sex or both sexes separately. The majority (98%) only had these experiences with members of the opposite sex. Generally, more intimate activities (from kissing to intercourse) increased with age.

**Table 3.** Selected frequencies across clinics of physical experiences with other young people of the opposite sex. (<5 may include zero). Note that generally if a YP answered ‘did not happen’, the questionnaire was curtailed at that point.

In the last year, YP has had this experience with/by a member of the opposite sex	F@11 (11.5yrs)	TF1 (12.5yrs)	TF2 (13.5yrs) *	TF3 (15.5yrs) *
	%	%	%	%
*Variable name*	*ferr026*	*ff5715*	*fg5147*	*fh8827*
Hugged	30.4	54.9	42.9	26.8
*Variable name*	*ferr030*	*ff5730*	*fg5157*	*fh8836*
Spent time alone together	21.6	39.2	28.7	28.8
*Variable name*	*ferr032*	*ff5740*	*fg5161*	*fh8841*
Kissed someone on lips	14.9	32.4	48.2	60.1
*Variable name*	*ferr034*	*ff5750*	*fg5171*	*fh8861*
Was kissed on lips	15.7	31.5	43.9	58.2
*Variable name*	*ferr036*	*ff5760*	*fg5181*	*fh8881*
Cuddled	10.7	33.9	44.6	57.5
*Variable name*	*ferr038*	*ff5770*	*fg5191*	*fh8901*
Laid down together	<5	11.6	20.7	44.9
*Variable name*	*ferr040*	*ff5780*	*fg5201*	*fh8921*
Was touched under clothes	<5	4.9	13.0	41.4
*Variable name*	*ferr042*	*ff5801*	*fg5221*	*fh8941*
YP touched another under clothes	<5	4.8	11.3	37.9
*Variable name*	*ferr044*	*ff5820*	*fg5241*	*fh8961*
YP undressed with another with private parts showing	<5	1.7	4.6	26.2
*Variable name*	*ferr046*	*ff5840*	*fg5261*	*fh9001*
YP has touched another’s private parts	<5	1.5	4.2	24.6
*Variable name*	*-*	*ff5860*	*fg5281*	*fh9041*
YP’s private parts touched by another		1.3	3.8	24.4
*Variable name*	*-*	*ff5880*	*fg5301*	*fh9061*
Had oral sex		0.8	2.6	19.9
*Variable name*	*ferr048*	*ff5900*	*fg5321*	*fh9101*
Had sexual intercourse	<5	0.6	2.0	16.3

-Not asked; *Actual wording is ‘with another person’ – other questions ask if same sex, opposite sex or both sexes. These frequencies refer only to the opposite sex in TF2 and TF3.

For those who answered a question positively, the YPs were then asked whether they wanted to experience that activity, whether they were coerced (‘was made to do this’) and whether they enjoyed the experience. Table 4 indicates that for the three TeenFocus clinics at least 92% of respondents had wanted selected sexual experiences. At least 80% had enjoyed genital fondling, having oral sex or sexual intercourse, but <80% enjoyed being undressed with another young person at all three time points.

**Table 4.** Frequencies of ‘wantedness’ and enjoyment in those who reported they had had selected experiences (<5 may include zero).

Experience within the previous year	TF1 12.5yrs %	TF2 13.5yrs %	TF3 15.5yrs %
Specified with	Opposite sex	Either sex	Either sex
*YP undressed with another with private * *parts showing*	*ff5822/ff5823/* *ff5828*	*fg5243/fg5244/* *fg5249*	*fh8963/fh8964/* *fh8970*
YP wanted this to happen	95.5	94.4	97.4
YP was made to do this – Yes	7.3	5.0	3.5
Enjoyed this experience – Yes *	77.7	75.5	79.3
*YP had touched/fondled another’s* * private parts*	*ff5842/ff5843* *ff5848*	*fg5263/fg5264/* *fg5269*	*fh9003/fh9004/* *fh9010*
YP wanted this to happen	97.0	96.6	98.2
YP was made to do this - Yes	12.2	6.5	2.8
Enjoyed this experience – Yes *	86.0	81.2	85.1
*YP’s private parts touched by another*	*ff5862/ff5867*	*fg5283/fg5288*	*fh9043/fh9049*
YP wanted this to happen	92.1	96.3	97.8
YP was made to do this - Yes	-	-	-
Enjoyed this experience – Yes *	87.8	85.1	90.4
*YP had oral sex*	*ff5882/ff5883/* *ff5888*	*fg5303/fg5304/* *fg5309*	*fh9063/fh9064/* *fh9071*
YP wanted this to happen	93.0	97.0	97.3
YP was made to do this - Yes	13.6	6.7	4.7
Enjoyed this experience – Yes *	91.2	87.8	87.9
*YP had sexual intercourse*	*ff5902/ff5903/* *ff5913*	*fg5323/fg5324/* *fg5334*	*fh9103/fh9104/* *fh9113*
YP wanted this to happen	97.6	98.4	97.8
YP was made to do this - Yes	<5	6.3	3.7
Enjoyed this experience – Yes *	97.6	93.0	95.6

*Combined: Yes, quite a lot/Yes, very much; YP = Young Person; - not asked

Table 5 gives the options for seven reasons for taking part in selected sexual activities in three TeenFocus clinics (and also for sexual intercourse at age 21). The reasons with the highest proportions were the natural progression of the relationship (at least 70%) and ‘loved this person’ (>57%). Low frequencies were reported for peer pressure (‘friends do it’) or ‘to avoid being dumped’.

**Table 5.** Reason(s) given for doing selected sexual experiences (percentages are of those who reported they have done this activity) (<5 may include zero) and multiple reasons may have been reported.

Experience within previous year	TF1 (12.5yrs) N (%)	TF2 (13.5yrs) N (%)	TF3 (15.5yrs) N (%)	21yrs+ N (%)	23yrs+ N (%)
Specified with	Opposite sex	Either sex	Either sex	Either sex	Either sex
*YP undressed by another with private* * parts showing*				-	-
Natural progression of relationship	80 (70.8)	233 (76.9)	1167 (75.2)		
Wanted to know what it was like	34 (30.1)	82 (27.1)	351 (22.6)		
Loved this person	83 (73.4)	181 (59.7)	920 (59.3)		
Friends do it	17 (15.0)	18 (5.9)	62 (4.0)		
To avoid being dumped/break-up	7 (6.2)	<5	12 (0.8)		
Got carried away	24 (21.2)	60 (19.8)	277 (17.8)		
Wanted to lose virginity	36 (31.8)	57 (18.8)	-	-	-
*YP has touched/fondled another’s* * private parts*				-	-
Natural progression of relationship	75 (75.0)	211 (80.8)	1080 (76.4)		
Wanted to know what it was like	33 (33.0)	76 (29.1)	346 (24.5)		
Loved this person	75 (75.0)	156 (59.8)	822 (58.2)		
Friends do it	16 (16.0)	23 (8.8)	47 (3.3)		
To avoid being dumped	<5	<5	13 (0.9)		
Got carried away	20 (20.0)	44 (16.9)	262 (18.5)		
Wanted to lose virginity	29 (29.0)	35 (13.4)	-	-	-
*YP’s private parts touched by another*					
Natural progression of relationship	67 (74.4)	195 (80.6)	1080 (77.0)	-	-
Wanted to know what it was like	27 (30.0)	75 (31.0)	370 (26.4)		
Loved this person	67 (74.4)	143 (59.1)	804 (57.3)		
Friends do it	14 (15.5)	14 (5.8)	49 (3.5)		
To avoid being dumped	<5	5 (2.1)	11 (0.8)		
Got carried away	16 (17.8)	39 (16.1)	248 (17.7)		
Wanted to lose virginity	25 (27.8)	35 (14.5)	-		
*YP has had oral sex*					
Natural progression of relationship	41 (71.9)	125 (75.8)	872 (77.0)	-	-
Wanted to know what it was like	23 (40.3)	52 (31.5)	345 (30.4)		
Loved this person	42 (73.7)	103 (62.4)	670 (59.1)		
Friends do it	10 (11.1)	7 (4.2)	26 (2.3)		
To avoid being dumped	<5	<5	10 (0.9)		
Got carried away	10 (11.1)	26 (15.8)	183 (16.1)		
Wanted to lose virginity	20 (35.1)	28 (17.0)	-		
*YP has had sexual intercourse*	*In last year*	*In last year*	*In last year*	*Most recent*	*Most recent*
Natural progression of relationship	30 (71.4)	94 (72.9)	719 (78.4)	2113 (72.3)	2673 (70.6)
Wanted to know what it was like	13 (30.9)	36 (27.9)	293 (31.9)	794 (27.1)	2365 (62.5)
Loved this person	35 (83.3)	83 (64.3)	623 (67.9)	1990 (70.0)	2562 (67.7)
Friends do it	<5	7 (5.4)	36 (3.9)	214 (7.3)	223 (5.9)
To avoid being dumped	<5	<5	10 (1.1)	21 (0.7)	35 (0.9)
Got carried away	6 (2.4)	24 (18.6)	142 (15.5)	316 (10.8)	293 (7.7)
Wanted to lose virginity	21 (50.0)	39 (30.2)	163 (17.8)	-	-
Because they wanted to	-	-	-	2842 (97.2)	3554 (93.9)
Sex work (sex exchanged for cash or other valuables)	-	-	-	5 (0.2)	<5
For another reason	-	-	-	131 (4.5)	82 (2.2)

-Not asked at that time point

Table 6 shows whether the respondents regretted selected sexual experiences for three TeenFocus clinics (and for sexual intercourse at 21+ and 23+ years). The actual question asked how much they had regretted the experience on a 4-point Likert scale: No regrets at all; Regret a bit; Regret quite a lot; Regret very much. Frequencies are shown of those experiencing the activity and what proportions had ‘no regrets at all’ versus combined ‘any regret’ to protect participant confidentiality where the numbers were small. As the respondents aged, the proportions saying they had any regrets decreased, for example, for sexual intercourse 31% expressed regret at 12.5 years versus 5% at age 23. 

**Table 6.** Frequencies of whether the YP regretted selected sexual experiences that occurred within the previous year (or on most recent occasion at ages 21+ and 23+ years).

Experience within previous year/ most recent occasion	TF1 12.5yrs (%)	TF2 13.5yrs (%)	TF3 15.5yrs (%)	21+yrs (%)	23+yrs (%)
Specified with	Opposite sex	Either sex	Either sex	Either sex	Either sex
*YP undressed with another with private* * parts showing*	*ff5836*	*fg5257*	*fh8979*	*-*	*-*
No regrets at all	66.1	70.1	82.2		
Combined regrets *	33.9	29.9	17.8		
*YP has touched/fondled another’s * *private parts*	*ff5860*	*fg5277*	*fh9019*	*-*	*-*
No regrets at all	71.0	78.5	85.9		
Combined regrets *	29.0	21.5	14.1		
*YP’s private parts touched by another*	*ff5875*	*fg5296*	*fh9058*	*-*	*-*
No regrets at all	75.6	82.2	87.4		
Combined regrets *	24.4	17.8	12.6		
*Had oral sex*	*ff5896*	*fg5317*	*fh9081*	*-*	*-*
No regrets at all	75.4	78.2	88.1		
Combined regrets *	24.6	21.8	11.9		
*Had sexual intercourse*	*ff5921*	*fg5342*	*fh9123*	*YPA3090*	*YPC0310*
No regrets at all	69.0	79.8	86.0	91.1	95.0
Combined regrets *	31.0	20.2	14.0	8.9	5.0

*Combined: Regret A bit/Quite a lot/Very much -Not asked at that point

From TF1 at 12.5 years, participants were asked if they had been drinking alcohol (with a follow-on question asking about their degree of intoxication) or had been using drugs prior to the sexual experience (only for sexual intercourse at age 21+ years). Numbers admitting alcohol or drug use are very small. Table 7 shows whether the YP had drunk any alcohol or used drugs for selected activities. In general, proportions drinking alcohol and using drugs increased with age, with the highest levels at age 21+ years.

**Table 7.** Alcohol or drug use prior to selected sexual experiences. Percentages are of total attendees/questionnaire completers. (<5 may include zero).

Experience	TF1 12.5yrs (%)	TF2 13.5yrs (%)	TF3 15.5yrs (%)	21+yrs (%)
Specified with	Opposite sex	Either sex	Either sex	Either sex
*Kissed someone on lips*				*-*
Alcohol	1.1	3.5	14.9	
Drugs use	0.3	0.5	2.0	
*Was kissed on lips*				*-*
Alcohol	1.1	3.0	10.8	
Drugs use	0.1	0.3	1.6	
*Cuddled*				*-*
Alcohol	0.5	1.0	4.0	
Drugs use	0.1	0.2	0.9	
*Laid down together*				*-*
Alcohol	0.5	1.2	4.6	
Drug use	0.1	0.3	0.8	
*Was touched under clothes*				*-*
Alcohol	0.4	1.3	6.4	
Drug use	0.1	0.2	0.9	
*YP touched another under clothes*				*-*
Alcohol	0.4	0.8	4.8	
Drug use	0.1	0.2	0.8	
*YP undressed with another with* * private parts showing*				*-*
Alcohol	0.2	0.6	3.5	
Drug use	<5	0.1	0.7	
*YP has touched another’s private parts*				*-*
Alcohol	0.1	0.6	3.1	
Drug use	<5	0.2	0.8	
*YP’s private parts touched by another*				*-*
Alcohol	0.1	0.4	3.0	
Drug use	<5	0.1	0.6	
*Had oral sex*				*-*
Alcohol	0.1	0.4	2.8	
Drug use	<5	0.1	0.5	
*Had sexual intercourse*				
Alcohol	<5	0.4	2.0	19.2
Drug use	<5	0.1	0.5	2.2

-Not asked at this time

One measure of sexual ‘readiness’ is whether young people carry condoms with them. Table 8 gives selected sexual experiences for which they were asked if they had carried a condom and, where appropriate, if a condom had been used. From TF1 (12.5 years) of those who had been undressed with another (with private parts showing), the number carrying condoms was almost 57% and over 73% at 15.5 years. Similar proportions are noted for the YP touching another’s genitals, YP’s genitals touched by another and having oral sex. But of the 73.6% who had a condom with them when they had had oral sex, only 16.7% said they had used one.

**Table 8.** Frequency of planned contraception use as one measure of readiness for sexual intercourse. Percentages are the proportion of those experiencing each activity.

Experience within previous year or for 21+yrs, on most recent occasion	TF1 12.5yrs (%)	TF2 13.5yrs (%)	TF3 15.5yrs (%)	TF4 17yrs * (%)	21+yrs (%)	23+yrs (%)
Specified with	Opposite sex	Either sex	Either sex	Either sex	Either sex	Either sex
*YP undressed with another with* * private parts showing*	*ff5827*	*fg5248*	*fh8969*	*-*	*-*	*-*
Had condom with them	56.8	64.0	73.1			
*YP has touched/fondled another’s private parts*	*ff5847*	*fg5268*	*fh9009*	*-*	*-*	*-*
Had condom with them	52.5	67.7	73.3			
*YP’s private parts touched by another*	*ff5866*	*fg5287*	*fh9048*	*-*	*-*	*-*
Had condom with them	57.3	62.1	72.1			
*Last occasion had oral sex*	*ff5883*	*fg5308*	*fh9069/* *fh9070*	*-*		*-*
Had condom with them	59.6	66.1	73.6			
Used a condom	-	-	16.7			
*Last occasion had sexual intercourse*	*ff5887*	*fg5328/* *fg5329*	*fh9109/* *fh9110*	*fjch707*	*YPA3070/* *YPA3080*	*YPC0270*
Used a condom	83.3	80.3	81.7	51.0	29.3	23.8
Used other form of contraception	23.8	26.0	41.1	-	66.8	67.2

-Not asked; *TF4 Chlamydia session

For the last occasion on which they had sexual intercourse, between TF1 and TF3 over 80% used a condom, but thereafter the proportions decrease to 51% at 17 years, 29.3% at 21 and 23.8% at 23. This may be due to the YP being in a committed relationship and using alternative forms of contraception or that they were planning to conceive. Roughly a quarter of YPs used alternative forms at TF1 and TF2, rising to 41.1% at TF3 and two-thirds at ages 21+ and 23+ years. This was not asked at TF4 (the Chlamydia sub-study).

Table 9a indicates the self-reported numbers of sexual partners up to the point of enquiry. These questions are inconsistent over time for reasons such as appropriateness to the respondents’ age for example: oral sex partners was only asked at TF3; sexual intercourse partners at TF1-TF3; number of sexual intercourse partners within the previous year and ever at TF1–TF4, 21+ and 23+ years; whether any sexual partners within the previous year were new at TF3 and TF4; whether the YP has ever had sexual intercourse with either a male or female partner at TF4, 21+ and 23+ years. The number of partners for questions has been categorised as follows: 1 partner, 2–5 and 6+ partners in order to protect confidentiality where numbers are small. As expected, a single sexual partner had higher frequencies at younger ages, with over 20% having had 2-5 partners from age 17. For those having 6 or more partners ever, this rose from ~7.5% at TF4 (17.5 years) to 33.9% and 42.7% at 21+ and 23+ years respectively.

**Table 9a.** For those reporting sexual intercourse, the numbers of sexual partners ever at each timepoint as a percentage of total clinic attendees/questionnaire completers (<5 may include zero).

	TF1 12.5yrs (%)	TF2 13.5yrs (%)	TF3 15.5yrs (%)	TF4 17yrs* (%)	21+yrs (%)	23+yrs (%)
Specified	Opposite sex	Either sex	Either sex	Either sex	Either sex	Either sex
*Oral sex*	-	-	*fh9065*	-	-	-
1 partner			10.6			
2-5 partners			7.5			
6+ partners			1.9			
*Sexual intercourse with:*	*ff5922*	*fg5343*	*fh9105*	-	-	-
1 partner	0.3	1.1	9.9			
2-5 partners	0.3	0.7	5.1			
6+ partners	<5	0.3	1.4			
*YP has ever had sexual* * intercourse (with either a female* * or male partner)*	*-*	*-*	*-*	*fjch700*	*YPA3022*	*YPC0210/ * *YPC0211*
Yes				49.5	94.6	97.0
No				28.5	5.4	3.0
*Sexual intercourse partners within* * last year:*	-	-	*fh9124*	*fjch704*	*YPA3110*	*YPC0340*
None			<5	2.1	5.2	<5
1 partner			10.6	28.7	51.6	58.8
2-5 partners			4.7	16.7	22.2	20.1
6+ partners			0.9	1.9	4.5	3.8
*Sexual intercourse partners ever*	-	-	*fh9126*	*fjch703*	*YPA3100*	*YPC0320*
None			<5	0.1	<5	<5
1 partner			9.4	19.1	16.7	13.3
2-5 partners			5.7	22.7	32.3	32.7
6+ partners			1.2	7.4	33.9	42.7
*Within last year, some sexual* * intercourse partners were new*	-	-	*fh9125*	*fjch708*	*-*	*-*
Yes			10.0	36.3		
No			6.1	12.6		

-Not asked

Table 9b gives the frequencies of the number of sexual partners in the previous 12 months and ever at ages 21+ years and 23+ years again, but this time split by sex assigned at birth. It can be seen that males had slightly higher rates of multiple partners in the previous year; with a similar pattern for numbers of sexual partners ever, with exceptions for 6-10, 11-20 and 21-30 sexual partners where females were slightly higher. The range of reported numbers of sexual partners are also given.

**Table 9b.** Of those reporting sexual intercourse in past year and ever, numbers of sexual intercourse partners split by assigned sex at birth as reported at 21+ years and 23+ years, along with the range.

	21+ years		23+ years	
Sex at birth:	Male	Female	Male	Female
*No. of sexual partners in past year:*	*YPA3110*		*YPC0340*	
1 partner	539 (61.5)	1249 (68.0)	754 (65.8)	1730 (73.6)
2-5 partners	265 (30.2)	503 (27.4)	313 (27.3)	537 (22.8)
6-10 partners	50 (5.7)	70 (3.8)	59 (5.1)	63 (2.7)
11+ partners	23 (2.6)	14 (0.8)	19 (1.6)	20 (0.8)
Total	877	1836	1145	2350
*Range*	*1-40*	*1-60*	*1-60*	*1-60*
*No. of sexual partners in lifetime:*	*YPA3100*		*YPC0320*	
1 partner	212 (22.1)	366 (19.1)	212 (16.8)	350 (14.1)
2-5 partners	395 (41.3)	724 (37.8)	476 (37.7)	905 (36.5)
6-10 partners	176 (18.4)	463 (24.2)	261 (20.7)	621 (25.0)
11-20	111 (11.6)	259 (13.5)	210 (16.6)	422 (17.0)
21-30	30 (3.1)	76 (4.0)	59 (4.7)	119 (4.8)
31+	33 (3.4)	26 (1.3)	45 (3.6)	64 (2.6)
Total	957	1914	1263	2481
*Range*	*1-150*	*1-300*	*1-300*	*1-210*

At the ages of 15.5, 21+ and 23+ years, the YPs were asked to indicate how they would describe their sexuality with options for 100% Heterosexual, Mostly heterosexual but also attracted to same sex, Bisexual, Mostly homosexual but also attracted to opposite sex, 100% Homosexual, Not attracted to either sex, Not sure, and at 23+ years only, ‘Other’. Table 10, split by assigned sex at birth of the YP, shows that the majority consider themselves heterosexual. Higher proportions of males stated they were homosexual (over 5% at the later ages).

**Table 10.** Young Person’s description of their sexuality by assigned sex at birth. (<5 may include zero).

Sexuality	TF3 (15.5yrs) N=5158		YPA 21+yrs N=3341		YPC 23+yrs N=4160	
Variable	*fh9140*		*YPA3000*		*YPC0420*	
Sex at birth (kz021):	Male	Female	Male	Female	Male	Female
100% Heterosexual	89.6	84.2	85.3	82.4	83.0	76.3
Mostly heterosexual but also attracted to same sex	6.0	10.7	5.9	12.4	7.5	16.7
Bisexual	1.2	2.0	1.2	2.2	1.2	2.9
Mostly homosexual but also attracted to opposite sex	0.6	0.5	1.4	1.1	1.4	1.1
100% Homosexual	0.7	<5	5.1	0.8	5.1	1.2
Not attracted to either sex	0.3	0.3	0.5	0.4	<5	0.3
Not sure	1.5	2.1	0.6	0.6	0.8	1.0
Other	-	-	-	-	0.9	0.6

-Not asked

Table 11 shows frequencies regarding the YP’s three most recent sexual partners at 21+ years of age. For the most recent partner, over 14% of females and 18% of males said they would definitely not have sex with them again and over half said they had used a condom on the first occasion. Over 14% believed that their most recent partner had had sex with someone else between the first and most recent sex together. Over 16% had reported overlap between their sexual partners.

**Table 11.** YPA 21+ years questionnaire frequencies concerning the respondents three most recent sexual partners, split by sex assigned at birth.

	Most recent partner		2 ^nd^ most recent partner		3 ^rd^ most recent partner	
Assigned sex at birth	Male	Female	Male	Female	Male	Female
*Gender of most recent sexual partners*	*YPA3122*		*YPA3142*		*YPA3162*	
Male	74 (7.7)	1877 (97.9)	64 (9.7)	1308 (97.4)	44 (9.2)	998 (97.6)
Female	885 (92.3)	41 (2.1)	595 (90.3)	35 (2.6)	436 (90.8)	24 (2.3)
Total	959	1918	659	1343	480	1022
*YP likely to have sex with this partner again*	*YPA3123*		*YPA3143*		*YPA3163*	
Yes	549 (57.3)	1365 (71.1)	27 (4.1)	46 (3.4)	10 (2.1)	22 (2.2)
Probably	79 (8.2)	110 (5.7)	29 (4.4)	44 (3.3)	30 (6.2)	21 (2.1)
Probably not	91 (9.5)	92 (4.8)	122 (18.4)	136 (10.1)	81 (16.8)	74 (7.3)
No	174 (18.2)	271 (14.1)	446 (67.4)	1054 (78.5)	329 (68.4)	861 (84.6)
Don’t know	65 (6.8)	81 (4.2)	38 (5.7)	62 (4.6)	31 (6.4)	40 (3.9)
Total	958	1919	662	1342	481	1018
*Most recent time YP had sex with this partner, was* * the 1 ^st^ occasion*	*YPA3124*		*YPA3144*		*YPA3164*	
Yes (only had sex with them once)	191 (20.0)	226 (11.8)	291 (44.1)	474 (35.3)	223 (46.5)	396 (39.1)
No, more than one occasion	766 (80.0)	1695 (88.2)	369 (55.9)	868 (64.7)	257 (53.5)	617 (60.9)
Total	957	1921	660	1342	480	1013
*YP used a condom on 1 ^st^ occasion with this partner*	*YPA3128*		*YPA3148*		*YPA3168*	
Yes	574 (60.0)	1043 (54.6)	393 (60.1)	771 (58.1)	286 (59.8)	608 (60.1)
No	306 (32.0)	790 (41.4)	211 (32.3)	499 (37.6)	149 (31.2)	361 (35.7)
Only had oral sex	76 (7.9)	77 (4.0)	50 (7.6)	57 (4.3)	43 (9.0)	43 (4.2)
Total	956	1910	654	1327	478	1012
*YP believes this partner had sex with another,* * between the 1 ^st^ and most recent sex together*	*YPA3130*		*YPA3150*		*YPA3170*	
Yes	137 (14.3)	272 (14.2)	136 (21.0)	298 (22.5)	104 (22.0)	203 (20.1)
Probably	53 (5.5)	90 (4.7)	101 (15.6)	162 (12.2)	62 (13.1)	123 (12.2)
Probably not	60 (6.3)	105 (5.5)	57 (8.8)	106 (8.0)	40 (8.5)	73 (7.2)
No	594 (62.1)	1304 (68.3)	195 (30.0)	455 (34.4)	135 (28.5)	358 (35.4)
Have only had sex once with this partner	108 (11.3)	123 (6.4)	154 (23.7)	293 (22.1)	129 (27.3)	248 (24.5)
Prefer not to say	5 (0.5)	16 (0.8)	6 (0.9)	9 (0.7)	<5	6 (0.6)
Total	957	1910	649	1323	473	1011
Overlap * between sexual partners	*N/A*		*YPA3152*		*YPA3172*	
Yes			122 (18.6)	215 (16.2)	85 (17.7)	193 (19.0)
No			509 (77.7)	1080 (81.6)	376 (78.2)	793 (78.0)
Not sure			18 (2.7)	21 (1.6)	18 (3.7)	25 (2.5)
Prefer not to say			6 (0.9)	8 (0.6)	<5	5 (0.5)
Total			655	1324	481	1016

*i.e. overlap between most recent and 2 ^nd^ most recent or between 2 ^nd^ and 3 ^rd^ most recent partners

Table 12a and Table 12b relate solely to data collected at 23+ years. Table 12a reports on sexual problems lasting more than three months within the past year including a lack of interest in sex (21.5%), lack of enjoyment, anxiety, pain, lack of arousal, erectile dysfunction (3.2%), lack of orgasm (24.4%) etc. About 36% did not experience any of these problems. Table 12b gives the frequencies of sources of advice for sexual problems, just over half did not seek any help at all, and just over 18% sought advice from friends or family.

**Table 12a.** YPC 23+ years questionnaire frequencies concerning problems with sexual functioning lasting 3 months or longer within the past year (N=4222).

Sexual Function	N (%)
*Has lacked interest in sex*	*YPC0350*
Yes	908 (21.5)
*Lacked enjoyment in sex*	*YPC0351*
Yes	521 (12.3)
*Has felt anxious during sex*	*YPC0352*
Yes	452 (10.7)
*Has felt physical pain as a result of sex*	*YPC0353*
Yes	449 (10.6)
*Has felt no excitement or arousal during sex*	*YPC0354*
Yes	351 (8.3)
*Failed to reach orgasm or took a long time to climax* * despite feeling excited/aroused*	*YPC0355*
Yes	1032 (24.4)
*Experience an orgasm more quickly than they would like*	*YPC0356*
Yes	358 (8.5)
*Women only – experienced an uncomfortably dry vagina*	*YPC0357*
Yes	401 (9.5)
*Men only – trouble getting/keeping an erection*	*YPC0358*
Yes	137 (3.2)
*Respondent did not experience any of the above problems*	*YPC0359*
Yes	1515 (35.9)

**Table 12b.** YPC 23+ years frequencies relating to sources of help and advice for sexual functioning problems. Percentages are of those completing the questionnaire. (<5 may include zero).

Question/ *variable*	%
*Respondent has sought help/advice regarding sex life from family or friends in last year*	*YPC0400*
Yes	18.3
*Respondent has sought help/advice regarding sex life from internet support sites/information*	*YPC0401*
Yes	12.0
*Respondent has sought help/advice regarding sex life from self-help books/leaflets in last year*	*YPC0402*
Yes	1.0
*Respondent has sought help/advice regarding sex life from self-help groups in last year*	*YPC0403*
Yes	<5
*Respondent has sought help/advice regarding sex life from a helpline in last year*	*YPC0404*
Yes	0.1
*Respondent has sought help/advice regarding sex life from their GP/family doctor in last year*	*YPC0405*
Yes	6.1
*Respondent has sought help/advice regarding sex life from sexual health/GUM/STI clinic in last year*	*YPC0406*
Yes	6.3
*Respondent has sought help/advice regarding sex life from psychiatrist or psychologist in past year*	*YPC0407*
Yes	0.8
*Respondent has sought help/advice regarding sex life from a relationship counsellor in past year*	*YPC0408*
Yes	0.3
*Respondent has sought help/advice regarding sex life from other type of clinic or doctor in past year*	*YPC0409*
Yes	0.7
*Respondent has sought help/advice regarding sex life from other help or advice source in past year*	*YPC0411*
Yes	0.8
*Respondent has **not** sought any help/advice regarding sex life in past year*	*YPC0410*
Yes	55.1

Table 13. By the 23+ years questionnaire, 28.6% of respondents had been either married, in a civil partnership or cohabiting as a couple for at least a year and there followed questions with options on a 5-point Likert scale from disagree strongly to agree strongly on: sexual compatibility (same interest in having sex), likes and dislikes, feeling emotionally close to their partner when having sex, satisfaction, concerns, avoiding sex because of sexual difficulties. The majority answered positively to questions on sharing the same interest levels, likes and dislikes, emotional closeness and sexual satisfaction. However, 14% felt distressed or worried about their sex life and over 11% avoided intercourse because of their own or their partner’s sexual difficulties.

**Table 13.** YPC 23+ years frequencies relating to respondents’ sexual functioning within cohabiting couples. Percentages are of those answering the question.

Sexual functioning	N (%)
*Married, civil partnership or cohabiting with a partner as a couple for 1+ years.*	*YPC0370*
No	2727 (71.4)
Yes	1093 (28.6)
Total	3820
*Both share about the same interest level in having sex*	*YPC0380*
Disagree strongly	47 (4.3)
Disagree	298 (27.3)
Neither agree nor disagree	69 (6.3)
Agree	403 (37.0)
Agree strongly	273 (25.0)
Total	1090
*Both share same sexual likes and dislikes*	*YPC0381*
Disagree strongly	7 (0.6)
Disagree	58 (5.3)
Neither agree nor disagree	125 (11.5)
Agree	565 (51.9)
Agree strongly	333 (30.6)
Total	1088
*Both experienced sexual difficulties in past year*	*YPC0382*
Disagree strongly	500 (46.2)
Disagree	333 (30.7)
Neither agree nor disagree	101 (9.3)
Agree	124 (11.4)
Agree strongly	25 (2.3)
Total	1083
*Respondent feels emotionally close to their partner when having sex together*	*YPC0383*
Hardly ever	10 (0.9)
Not very often	15 (1.4)
Sometimes	91 (8.4)
Most of the time	326 (30.1)
Always	642 (59.2)
Total	1084
*Respondent feels satisfied with their sex life in last year*	*YPC0390*
Disagree strongly	176 (4.3)
Disagree	575 (13.9)
Neither agree nor disagree	676 (16.3)
Agree	1517 (36.7)
Agree strongly	1191 (28.8)
Total	4135
*Respondent feels distressed or worried about their sex life in last year*	*YPC0391*
Disagree strongly	1499 (36.4)
Disagree	1326 (32.2)
Neither agree nor disagree	713 (17.3)
Agree	495 (12.0)
Agree strongly	82 (2.0)
Total	4115
*Respondent has avoided sex because of sexual difficulties (their own or those of their partner)*	*YPC0392*
Disagree strongly	2127 (51.9)
Disagree	1071 (26.2)
Neither agree nor disagree	422 (10.3)
Agree	388 (9.5)
Agree strongly	87 (2.1)
Total	4095

## What has been found so far

Some of this extensive longitudinal data have already been analysed, but there is scope for much more:

A paper examining these data found that 17% of 11- to 12-year-olds and 32% of 12- to 13-year-olds reported having been kissed on the mouth. A minority of 12- to 13-year-olds reported sexual behaviour including intercourse, and the majority reporting such behaviour did not express regret. Boys especially, were less likely to regret it and reported all activities more than girls ( Waylen *et al.*, 2010).

Heron *et al.* (2015) aimed to describe rates of early coitarche (<16 years) and sexual ‘readiness’ in adolescence and to assess the extent to which these were socially patterned. Using the TF3 (15.5-year data), they found that 17.7% of respondents reported sexual intercourse in the previous 12 months. Numbers with sexual experiences were higher in girls (RR 1.30). About a third of the 15-year-olds were classed as ‘unready’ as measured by being unwilling, lacking in autonomy, regretting the experience or not using contraception. They found no social patterning in sexual readiness.

Van Leeuwen and Mace (2016) examined life history factors, personality and the social clustering of sexual experience in adolescents at age 17.5 years. They found substantial clustering within friendship networks, but minimal clustering at the school and neighbourhood level, which suggested a limiting role of socio-ecological influences at those levels. After accounting for personality and life history factors there remained a substantial unexplained similarity among friends. They interpreted this as either a tendency to associate with similar individuals or the social transmission of behavioural norms.

Xu *et al.* (2019) tested the association between multiple prenatal and postnatal early life factors and adolescent sexual orientation in 5007 ALSPAC teenagers. They controlled for childhood gender non-conformity (GNC), handedness, and digit ratio as markers of prenatal androgen exposure. They found that GNC was strongly associated with later male and female non-heterosexuality, and higher right-hand digit ratio was associated with later male non-heterosexuality. In the boys, later non-heterosexual orientation was more likely if they had a low birth weight and shorter breastfeeding duration. In girls, parental absence since birth, low prenatal family socioeconomic position, and poorer parent–child relationship were all associated with later non-heterosexuality. Following on from this study, Xu *et al.* (2021a) quantified changes in self-reported sexual orientation from adolescence to early adulthood and investigated whether GNC predicted sexual orientation changes measured at ages 15.5, 21, and 23 years. They concluded that self-reported sexual orientation from adolescence to young adulthood was more stable in males compared with females.

Another paper by Xu *et al.* (2021b) aimed to examine sexual behaviour patterns, the consistency between sexual behaviour and sexual orientation using the data collected at 13.5 and 15.5 years. Latent class analyses suggested four subgroups: (i)“high-intensity sexual behaviours exclusively with other-sex, no same-sex intimacy”; (ii) “moderate-intensity sexual behaviours exclusively with other-sex, no same-sex intimacy”; (iii) “low-intensity sexual behaviours exclusively with other-sex, no same-sex intimacy”; and (iv) “no sexual behaviour”. At 15.5 years, there were the four sub-groups as at 13.5 plus a new group (v) “high-intensity sexual behaviours, some same-sex intimacy” (5.6%). They found moderate consistencies between same-sex intimacy and same-sex attraction for boys and low consistency for girls and suggested that “it may be important to include low-intensity sexual behaviours when assigning adolescents to sexual orientation groupings (via sexual behaviours) as this would reduce selection biases and increase statistical power via the increase in sample size”.

A study by Camenga *et al.* (2023) found that oral contraceptive use <17 years was associated with higher odds of several lower urinary tract (UT) symptoms at 19, and these associations were attenuated after adjustment for the number of sexual partners and condom use. Increased odds of all lower UT symptoms (but not stress incontinence) were associated with having more than three sexual partners by the age of 17.

Readers may find the data note on puberty within ALSPAC helpful when using these data ( Golding *et al.*, 2023). In brief, puberty questionnaires were administered between the ages of 8 to 18 years and used the Tanner scales, as well as direct questions on key milestones such as menarche and voice-breaking, height and weight.

## Strengths and limitations

A key strength of these data is that the population is geographically defined and included the majority (~80%) of the eligible pregnant population at the time of enrolment. Other strengths include a large sample size, the longitudinal design and regular administration of questionnaires and yearly face-to-face clinics that reduce the chance of recall bias, with additional availability of data on potential confounding factors.

As with all longitudinal studies, attrition is a problem which is strongly associated with socioeconomic disadvantage. At the time of enrolment there were very few families of non-white residents in the Avon area (~6%) and therefore research results cannot be extrapolated to cover non-white populations in general.

In the case of the offspring cohort, more females remain committed to the study than males. For these data on sexual experiences, it would be prudent to consider the respondents’ honesty in answering sensitive questions with the potential for under-reporting as well as exaggeration (for example, outliers in number of sexual partners). In the clinic settings, the tester recorded the demeanour of the YP (e.g. shy) if the respondent terminated the interview or refused to start.

A further limitation was the computerised system used in the clinics did not ask subsequent questions if the answer was No (e.g. if the YP and their friend had NOT spent time alone together, then all subsequent questions were not asked).

## Ethics and consent

Ethical approval for the ALSPAC study was obtained from the ALSPAC Ethics and Law Committee (ALEC; IRB00003312) and the Local Research Ethics Committees. Detailed information on the ways in which confidentiality of the cohort is maintained may be found on the study website: http://www.bristol.ac.uk/alspac/researchers/research-ethics/. All methods were performed in accordance with the relevant guidelines and regulations at the time. Informed consent for the use of data collected via questionnaires was assumed from participants following the recommendations of the ALSPAC Ethics and Law Committee at the time ( Birmingham, 2018). Signed consent was obtained for information obtained in the hands-on clinics.

## Data Availability

ALSPAC data access is through a system of managed open access. The steps below highlight how to apply for access to the data included in this data note and all other ALSPAC data: •   1. Please read the ALSPAC access policy ( http://www.bristol.ac.uk/media-library/sites/alspac/documents/researchers/data-access/ALSPAC_Access_Policy.pdf) which describes the process of accessing the data and samples in detail, and outlines the costs associated with doing so. •   2. You may also find it useful to browse our fully searchable research proposals database ( https://proposals.epi.bristol.ac.uk/?q=proposalSummaries), which lists all research projects that have been approved since April 2011. •   3. Please submit your research proposal ( https://proposals.epi.bristol.ac.uk/) for consideration by the ALSPAC Executive Committee. You will receive a response within 10 working days to advise you whether your proposal has been approved. Open Science Framework: Sexual experiences and behaviours in the offspring generation of the Avon Longitudinal Study of Parents and Childhood. https://doi.org/10.17605/OSF.IO/486W2 ( Iles-Caven, 2024). This project contains the following extended data: Supplementary Tables.docx. (Variable names). Data are available under the terms of the Creative Commons Attribution 4.0 International license (CC-BY 4.0).

## References

[ref-1] Ary DV Duncan TE Duncan SC : Adolescent problem behavior: the influence of parents and peers. *Behav Res Therapy.* 1999;37(3):217–230. 10.1016/s0005-7967(98)00133-8 10087640

[ref-2] Birmingham K : Pioneering ethics in longitudinal studies: The early development of the ALSPAC ethics & law committee.Bristol: Policy Press,2018. 10.1332/9781447340423

[ref-3] Boyd A Golding J Macleod J : Cohort Profile: the 'children of the 90s'--the index offspring of the Avon Longitudinal Study of Parents and Children. *Int J Epidemiol.* 2013;42(1):111–127. 10.1093/ije/dys064 22507743 PMC3600618

[ref-4] Brooks-Gunn J Furstenberg FF Jr : Adolescent sexual behavior. *Am Psychol.* 1989;44(2):249–257. 10.1037/0003-066X.44.2.249 2653137

[ref-5] Camenga DR Wang Z Chu H : Sexual health behaviors by age 17 and Lower Urinary Tract Symptoms at age 19: PLUS research consortium analysis of ALSPAC Data. *J Adolesc Health.* 2023;72(5):737–745. 10.1016/j.jadohealth.2022.12.019 36781327 PMC10826680

[ref-6] Crichton J Hickman M Campbell R : Prevalence of chlamydia in young adulthood and association with life course socioeconomic position: birth cohort study. *PLoS One.* 2014;9(8): e104943. 10.1371/journal.pone.0104943 25153124 PMC4143219

[ref-13] Fergusson DM Horwood LJ Lynskey MT : The comorbidities of adolescent problem behaviors: a latent class model. *J Abnorm Child Psychol.* 1994;22(3):339–354. 10.1007/BF02168078 8064037

[ref-7] Fraser A Macdonald-Wallis C Tilling K : Cohort Profile: the Avon Longitudinal Study of Parents and Children: ALSPAC mothers cohort. *Int J Epidemiol.* 2013;42(1):97–110. 10.1093/ije/dys066 22507742 PMC3600619

[ref-8] Golding J Iles-Caven Y Northstone K : Measures of puberty in the Avon Longitudinal Study of Parents and Children (ALSPAC) offspring cohort [version 2; peer review: 1 approved; 2 approved with reservations]. *Wellcome Open Res.* 2023;8:453. 10.12688/wellcomeopenres.19793.2 38716046 PMC11075130

[ref-9] Hansen WB Paskett ED Carter LJ : The Adolescent Sexual Activity Index (ASAI): a standardized strategy for measuring interpersonal heterosexual behaviors among youth. *Health Educ Res.* 1999;14(4):485–490. 10.1093/her/14.4.485 10557519

[ref-10] Harris PA Taylor R Thielke R : Research electronic data capture (REDCap)--a metadata-driven methodology and workflow process for providing translational research informatics support. *J Biomed Inform.* 2009;42(2):377–381. 10.1016/j.jbi.2008.08.010 18929686 PMC2700030

[ref-11] Herbert A Heron J Barter C : Risk factors for intimate partner violence and abuse among adolescents and young adults: findings from a UK population-based cohort [version 3; peer review: 2 approved]. *Wellcome Open Res.* 2021;5:176. 10.12688/wellcomeopenres.16106.3 33553678 PMC7848855

[ref-12] Heron J Low N Lewis G : Social factors associated with readiness for sexual activity in adolescents: a population-based cohort study. *Arch Sexual Behav.* 2015;44(3):669–678. 10.1007/s10508-013-0162-5 23982565

[ref-14] Houtepen LC Heron J Suderman MJ : Adverse childhood experiences in the children of the Avon Longitudinal Study of Parents and Children (ALSPAC) [version 1; peer review: 3 approved]. *Wellcome Open Res.* 2018;3:106. 10.12688/wellcomeopenres.14716.1 30569020 PMC6281007

[ref-16] Iles-Caven Y : Sexual experiences and behaviours in the offspring generation of the Avon Longitudinal Study of Parents and Childhood. OSF. [Dataset].2024. 10.17605/OSF.IO/486W2 PMC11574334 39563947

[ref-15] Iles-Caven Y Golding J : Sexual experiences, behaviours and attitudes in the parents in the Avon Longitudinal Study of Parents and Childhood (ALSPAC) [version 1; peer review: awaiting peer review]. *Wellcome Open Res.* 2024. 10.12688/wellcomeopenres.21263.2 PMC11574334 39563947

[ref-17] James J Ellis BJ Schlomer GL : Sex-specific pathways to early puberty, sexual debut, and sexual risk taking: tests of an integrated evolutionary-developmental model. *Dev Psychol.* 2012;48(3):687–702. 10.1037/a0026427 22268605

[ref-18] Jessor R : New perspectives on adolescent risk behaviour.Cambridge: Cambridge University Press,1998. 10.1017/CBO9780511571138

[ref-19] Kaestle CE Halpern CT Miller WC : Young age at first sexual intercourse and sexually transmitted infections in adolescents and young adults. *Am J Epidemiol.* 2005;161(8):774–780. 10.1093/aje/kwi095 15800270

[ref-20] Kahn NF Halpern CT : Associations between patterns of sexual initiation, sexual partnering, and sexual health outcomes from adolescence to early adulthood. *Arch Sex Behav.* 2018;47(6):1791–1810. 10.1007/s10508-018-1176-9 29594701 PMC6501817

[ref-21] Kotchick BA Shaffer A Forehand R : Adolescent sexual risk behavior: a multi-system perspective. *Clin Psychol Rev.* 2001;21(4):493–519. 10.1016/s0272-7358(99)00070-7 11413865

[ref-22] Lawn RB Anderson EL Suderman M : Psychosocial adversity and socioeconomic position during childhood and epigenetic age: analysis of two prospective cohort studies. *Hum Mol Genet.* 2018;27(7):1301–1308. 10.1093/hmg/ddy036 29365106 PMC5985722

[ref-23] Lussier AA Zhu Y Smith BJ : Association between the timing of childhood adversity and epigenetic patterns across childhood and adolescence: findings from the Avon Longitudinal Study of Parents and Children (ALSPAC) prospective cohort. *Lancet Child Adolesc Health.* 2023;7(8):532–543. 10.1016/S2352-4642(23)00127-X 37327798 PMC10527482

[ref-24] Mårdh PA Creatsas G Guaschino S : Correlation between an early sexual debut, and reproductive health and behavioral factors: a multinational European study. *Eur J Contracept Reprod Health Care.* 2000;5(3):177–182. 10.1080/13625180008500396 11131782

[ref-25] Mars B Heron J Crane C : Differences in risk factors for self-harm with and without suicidal intent: findings from the ALSPAC cohort. *J Affect Disord.* 2014;168:407–414. 10.1016/j.jad.2014.07.009 25108277 PMC4160300

[ref-27] Northstone K Lewcock M Groom A : The Avon Longitudinal Study of Parents and Children (ALSPAC): an update on the enrolled sample of index children in 2019 [version 1; peer review: 2 approved]. *Wellcome Open Res.* 2019;4:51. 10.12688/wellcomeopenres.15132.1 31020050 PMC6464058

[ref-28] Palmer MJ Clarke L Ploubidis GB : Is “Sexual Competence” at first heterosexual intercourse associated with subsequent sexual health status? *J Sex Res.* 2017;54(1):91–104. 10.1080/00224499.2015.1134424 26891245 PMC5214675

[ref-29] Sandfort TGM Orr M Hirsch JS : Long-term health correlates of timing of sexual debut: results from a national US study. *Am J Public Health.* 2008;98(1):155–161. 10.2105/AJPH.2006.097444 18048793 PMC2156059

[ref-30] Sidebotham P , ALSPAC Study Team : Patterns of child abuse in early childhood, a cohort study of the ‘Children of the Nineties’. *Child Abuse Rev.* 2000;9(5):311–320. 10.1002/1099-0852(200009/10)9:5<311::AID-CAR627>3.0.CO;2-U

[ref-31] Sidebotham P Golding J , ALSPAC Study Team : Child maltreatment in the “Children of the Nineties” A longitudinal study of parental risk factors. *Child Abuse Negl.* 2001;25(9):1177–1200. 10.1016/s0145-2134(01)00261-7 11700691

[ref-32] Sidebotham P Heron J Golding J : Child maltreatment in the “Children of the Nineties:” deprivation, class, and social networks in a UK sample. *Child Abuse Negl.* 2002;26(12):1243–1259. 10.1016/s0145-2134(02)00415-5 12464299

[ref-33] Sprecher S O’Sullivan LF Drouin M : The significance of sexual debut in women’s lives. *Curr Sex Health Rep.* 2019;11:265–273. 10.1007/s11930-019-00228-5

[ref-34] Troy D Russell A Kidger J : Childhood psychopathology mediates associations between childhood adversities and multiple health risk behaviours in adolescence: analysis using the ALSPAC birth cohort. *J Child Psychol Psychiatry.* 2021;62(9):1100–1109. 10.1111/jcpp.13379 33619761 PMC8532527

[ref-35] van Leeuwen AJ Mace R : Life history factors, personality and the social clustering of sexual experience in adolescents. *R Soc Open Sci.* 2016;3(10): 160257. 10.1098/rsos.160257 27853543 PMC5098968

[ref-36] Waylen AE Ness A McGovern P : Romantic and sexual behavior in young adolescents: Repeated surveys in a population-based cohort. *J Early Adolesc.* 2010;30(3):432–443. 10.1177/0272431609338179

[ref-37] Wellings K Jones KG Mercer CH : The prevalence of unplanned pregnancy and associated factors in Britain: findings from the third National Survey of Sexual Attitudes and Lifestyles (Natsal-3). *Lancet.* 2013;382(9907):1807–1816. 10.1016/S0140-6736(13)62071-1 24286786 PMC3898922

[ref-38] Xu Y Norton S Rahman Q : Early life conditions and adolescent sexual orientation: a prospective birth cohort study. *Dev Psychol.* 2019;55(6):1226–1243. 10.1037/dev0000704 30762413

[ref-39] Xu Y Norton S Rahman Q : Childhood gender nonconformity and the stability of self-reported sexual orientation from adolescence to young adulthood in a birth cohort. *Dev Psychol.* 2021a;57(4):557–569. 10.1037/dev0001164 33683915

[ref-40] Xu Y Norton S Rahman Q : Adolescent sexual behavior patterns in a British birth cohort: a latent class analysis. *Arch Sex Behav.* 2021b;50(1):161–80. 10.1007/s10508-019-01578-w 31907696 PMC7878235

