# Supplementary material for: Sexual experiences and behaviours in the offspring generation of the Avon Longitudinal Study of Parents and Childhood
Source: Wellcome Open Res. 2024 Nov 5;9:241. Originally published 2024 May 9. [Version 2] doi: 10.12688/wellcomeopenres.21687.2 (PMC11602696; doi:10.12688/wellcomeopenres.21687.2)
Supplement: Peer review history

# Reviewer report: Reviewer response for version 2

**DOI:** 10.21956/wellcomeopenres.25760.r114270

**Published:** 2024-12-20

**Authors:** Elise Bragard

**Affiliations:** 1 University of Connecticut School of Medicine, Farmington, USA

Thank you for the opportunity to review this data note. This data note describes longitudinal data on the romantic and sexual experiences of youth in the ALSPAC from pre-adolescence to adulthood, including specific behaviors at each time point and the degree of coercion/wantedness, condom/contraceptive use, and substance use associated with specific behaviors and experiences. The data is thorough and clearly described. My only recommendation is to carefully proofread the paper for grammar and punctuation issues that make writing unclear at times, e.g., in the first paragraph: check subject/verb agreement between “numbers” and “has,” resolve sentence fragments like those starting with “As well as,” etc. In the romantic relationships section: awkward phrasing like “on enjoyed the experience,” inconsistency in tense use, such as “respondents were asked” vs. “questions elicit details.”

Are sufficient details of methods and materials provided to allow replication by others?

Yes

Is the rationale for creating the dataset(s) clearly described?

Yes

Are the datasets clearly presented in a useable and accessible format?

Yes

Are the protocols appropriate and is the work technically sound?

Yes

Reviewer Expertise:

Developmental psychology, adolescent sexual health and development, substance use

I confirm that I have read this submission and believe that I have an appropriate level of expertise to confirm that it is of an acceptable scientific standard.

# Reviewer report: Reviewer response for version 2

**DOI:** 10.21956/wellcomeopenres.25760.r109891

**Published:** 2024-11-27

**Authors:** Habeeb Omoponle Adewuyi

**Affiliations:** 1 Departmentof Educational Psychology, University of Johannesburg, Auckland Park, Gauteng, South Africa

I have checked and the corrections have been implemented. The article can be accepted.

Are sufficient details of methods and materials provided to allow replication by others?

Yes

Is the rationale for creating the dataset(s) clearly described?

Yes

Are the datasets clearly presented in a useable and accessible format?

Partly

Are the protocols appropriate and is the work technically sound?

Yes

Reviewer Expertise:

Adolescent sexuality and psychology

I confirm that I have read this submission and believe that I have an appropriate level of expertise to confirm that it is of an acceptable scientific standard.

# Reviewer report: Reviewer response for version 1

**DOI:** 10.21956/wellcomeopenres.23960.r83961

**Published:** 2024-09-02

**Authors:** Habeeb Omoponle Adewuyi

**Affiliations:** 1 Departmentof Educational Psychology, University of Johannesburg, Auckland Park, Gauteng, South Africa

The article titled “Sexual experiences and behaviours in the offspring generation of the Avon Longitudinal Study of Parents and Childhood”

**Comments:** Although the topic addressed is relevant, limitations have been identified that significantly undermine the quality and clarity of the work.

**Introduction**

**Comments:**

 The introduction requires reorganization to achieve a clearer and more structured exposition. It is suggested that the authors define precisely, but uniquely, the concept of Sexual experiences and behaviours.

 You are encouraged to support claims about the relationship between variables (Sexual experiences and behaviours) with citations of empirical evidence. It is observed that sometimes the content of the Introduction is not consistent with the topic of study.

  - Address the structure of the introduction to ensure coherence and relevance.

**Structure of the Method**

**Comments:**

 The information in the "Method" section should be organized into traditional sections, and subsections of a scientific report. Specifically, authors should separate information on Participants, Procedure, and Data. The detailed description of the instruments must follow APA standards. For example, you must include the sociodemographic data of the sample.

 The inclusion of a specific section for "Statistical Analysis" will be applauded, detailing the methods and tests used before presenting the results is important especially stating the level of significance as 0.05 or as applicable.

**Results**

**Comments:**

 The results section has presented statistical data directly related to the research, thus improving interpretation for the reader.

**Discussion**

**Comments:**

 The discussion section should be approached after a thorough review of the literature related to the topic in the introduction. This will allow for more precise conclusions in the context of the research, highlighting the significant contribution of the research to the study of sexuality.

**Conclusion**

**Comments: **This section is poorly written and needs to be reconsidered.

 I encourage authors to maintain their effort and motivation, hoping that these suggestions will contribute to the future success of their research in a journal.

Are sufficient details of methods and materials provided to allow replication by others?

Yes

Is the rationale for creating the dataset(s) clearly described?

Yes

Are the datasets clearly presented in a useable and accessible format?

Partly

Are the protocols appropriate and is the work technically sound?

Yes

Reviewer Expertise:

Adolescent sexuality and psychology

I confirm that I have read this submission and believe that I have an appropriate level of expertise to confirm that it is of an acceptable scientific standard, however I have significant reservations, as outlined above.

# Response

**Published:** 2024-10-30

**Authors:** Yasmin Iles-Caven

**Affiliations:** Bristol Medical School: Population Health Sciences, University of Bristol, Bristol, UK

**Thank you for your useful comments on this paper.  Please note this paper does not contain analyses, it is purely a Data Note describing all the sexual behaviour/experiences measured in the ALSPAC cohort over time.  It is usual for data notes not to undertake a full literature review, but merely a brief background reasoning for why particular data were collected at the time.  However, in light of your comments and that of Reviewer 1, the Background has been rewritten slightly.** **“In order to better understand normal sexual development and behaviours, the Avon Longitudinal Study of Parents and Children (ALSPAC): (a) collected annual data on the stage of puberty attained between ages 8 and 17 (see Golding et al., 2023), and (b) administered questions on sexual experiences known as ‘Romantic relationships’ to the index offspring from the age of 11.5 years. The latter are described in this data note. In addition, we describe data concerning sexual experiences collected when they were 21 and 23 years of age. The early data sweeps at 11 and 12 were heavily focussed on heterosexual experiences.”** **Structure of the Method**

**Comments:**

 The information in the "Method" section should be organized into traditional sections, and subsections of a scientific report. Specifically, authors should separate information on Participants, Procedure, and Data. The detailed description of the instruments must follow APA standards. For example, you must include the sociodemographic data of the sample.

 The inclusion of a specific section for "Statistical Analysis" will be applauded, detailing the methods and tests used before presenting the results is important especially stating the level of significance as 0.05 or as applicable.

**Response: As mentioned above, this is not an analysis paper and therefore a description of statistical analyses is not warranted.  We are merely describing data collected in ALSPAC to enable interested researchers to understand the depth and breadth of such data.  These data will be of interest to epidemiologists as well as psychologists and have followed requirements for WOR rather than APA standards.  Sociodemographics are described in Table 1 for the whole cohort (first born/twin/ ethnicity, social class, housing tenure, urban/rural and IMD are based on the mothers’ report from questionnaires completed when pregnant with the study child.  Education, relationship status, living with a partner and currently a parent, were asked of the study ‘child’ at ages 26 and 29 –consequently numbers are less than the maternal figures in pregnancy due to attrition (as evidenced from Table 2). The intention is to give a broad description of the population.** 

**Results Comments:**

 The results section has presented statistical data directly related to the research, thus improving interpretation for the reader.

**Response: We labelled the ‘Results’ section ‘Observations’ as we were not reporting results of statistical analyses.**

****

**Discussion Comments:**

 The discussion section should be approached after a thorough review of the literature related to the topic in the introduction. This will allow for more precise conclusions in the context of the research, highlighting the significant contribution of the research to the study of sexuality.

**Conclusion**

**Comments: **This section is poorly written and needs to be reconsidered.  I encourage authors to maintain their effort and motivation, hoping that these suggestions will contribute to the future success of their research in a journal.

**Response: As WOR dictates, a data note includes no extensive literature review. Nor are authors required to provide a Discussion and Conclusion. Apart from briefly summarising the research that has already been undertaken using these data and the Strengths & Limitations of the data.**

**Thank you for your encouragement – we will certainly be analysing these data soon.**

# Reviewer report: Reviewer response for version 1

**DOI:** 10.21956/wellcomeopenres.23960.r85532

**Published:** 2024-06-28

**Authors:** Ashling Bourke

**Affiliations:** 1 School of Human Development, Institute of Education, Dublin City University, Dublin, Leinster, Ireland

This is a data-note on a very necessary longitudinal study on the sexual experiences of adolescents. It has the advantages of a large data set using well-constructed measures to explore sexuality development and experiences from adolescence through to early adulthood. The study is well designed, and the findings are provided in a clear manner. Given the preponderance of cross-sectional data in this field the availability of longitudinal data is much warranted.

 Some suggestions and recommendations:

 The research on age of first sexual experiences and impacts of same are quite dated. In order to situate your research in the current context, I would suggest citing papers from the past 10 years.   

 When describing the study and sample, it would be useful to give an overview of what wave of the study does this research pertain to – including the year the data was collected. It seems that the participants are around 29 years old- it would be good for the reader to have a sense of the time period when they were growing up. If the first waves of data for the study were collected  around 17 years ago, then more information on that context would be useful. For example, what would relationships and sexuality education or sexual health initiatives been like for this cohort of participants during the periods of data collection.

 It would also be useful to give a sense of when the various variables were collected. Table 1 has 1993 beside the rurality variable and 2014 beside the maternal deprivation variable – does indicate that the data for these variables were derived from different waves of the data?

 For Focus at 11, TF1-TF3 - Given that only the data pertaining to sexual activity with a member of the opposite sex, it would be important to highlight from early on, that the focus is on heterosexual sexual development and activity for these waves of the study. It should be made clear whether it was only these 1548 children that were tracked through for the adolescent years.

 It would be useful to provide the n and inclusion and exclusion criteria for each section. There seems to be a larger n for the adult data. If there were celibate participants (and asexual participants for the sexual orientation variables) were they included in the analysis.

 There is a limitation on the generalizability of a predominantly white sample - 95%- which is noted. There has been a significant demographic shift since the data were collected. This could be highlighted when noting the time-period in which the data was collected.

 I would suggest including the limited focus on heterosexual relationships and development in the limitations of the data.

Are sufficient details of methods and materials provided to allow replication by others?

Yes

Is the rationale for creating the dataset(s) clearly described?

Yes

Are the datasets clearly presented in a useable and accessible format?

Yes

Are the protocols appropriate and is the work technically sound?

Yes

Reviewer Expertise:

Sexuality education, sexual health, children's rights

I confirm that I have read this submission and believe that I have an appropriate level of expertise to confirm that it is of an acceptable scientific standard.

# Response

**Published:** 2024-10-30

**Authors:** Yasmin Iles-Caven

**Affiliations:** Bristol Medical School: Population Health Sciences, University of Bristol, Bristol, UK

This is a data-note on a very necessary longitudinal study on the sexual experiences of adolescents. It has the advantages of a large data set using well-constructed measures to explore sexuality development and experiences from adolescence through to early adulthood. The study is well designed, and the findings are provided in a clear manner. Given the preponderance of cross-sectional data in this field the availability of longitudinal data is much warranted.  Some suggestions and recommendations:

 The research on age of first sexual experiences and impacts of same are quite dated. In order to situate your research in the current context, I would suggest citing papers from the past 10 years.   

**Response:** **Thank you for your useful critique of our paper.** **We have slightly rewritten the background adding some more up-to-date references and removing some older references:** “The median age of sexual debut in Britain is 16, and the numbers reporting their debut before the age of 16 has been increasing over time (from 1 in 7 men/1 in 9 women born around 1950 to 1 in 3 in both sexes for those born around 1990) (Spiegelhalter, 2015 reporting on Natsal-3 data).  More than 25% of adolescents in the UK report sexual activity before age 16 ( Jessor, 1998) with mean age of sexual debut (coitarche or age at first intercourse) at 14 years for both boys and girls ( Brooks-Gunn & Furstenberg, 1989).  Adolescents are at higher risk of various negative health consequences associated with early coitarche and unsafe sexual activity, including sexually transmitted diseases (STIs) and unintended pregnancy (see Kotchick *et al.*, 2001 for a review; Mårdh *et al.*, 2000).  For example, early sexual debut has been found to be associated with non-volitional sex, termination of pregnancy and/or pregnancy loss, as well as infections of the reproductive tract (Peragine et al., 2022). Results from the UK’s NATSAL-3 report that over 45% of pregnancies occurring between ages 16–19 were unplanned and that coitarche <16 years was one strongly associated risk factor (aged adjusted OR 2.85) ( Wellings *et al.*, 2013).  ‘Sexual competence’ at sexual debut (defined as fulfilling four criteria: contraceptive protection; autonomy of decision (not due to external influences); that both partners were “equally willing”; and that it happened at the “right time” was found to be less likely in younger adolescents ( Palmer *et al.*, 2017). In addition, romantic and sexual relationships that begin at young ages are associated with an increased risk of maladaptive outcomes such as lower educational and occupational attainment and other risky behaviour such as alcohol and drug use (e.g. Ary *et al.*, 1999; Fergusson *et al.*, 1994; James *et al.*, 2012; and Sprecher *et al.*, 2019 for a review); and negative influences on mental health, relationship skills and sexual functioning in later life (e.g. Kahn & Halpern, 2018; Sandfort *et al.*, 2008)).”

 When describing the study and sample, it would be useful to give an overview of what wave of the study does this research pertain to – including the year the data was collected. It seems that the participants are around 29 years old- it would be good for the reader to have a sense of the time period when they were growing up. If the first waves of data for the study were collected  around 17 years ago, then more information on that context would be useful. For example, what would relationships and sexuality education or sexual health initiatives been like for this cohort of participants during the periods of data collection.

**Response: We have added dates for each data sweep in the 2 ^nd^ paragraph of the Methods section and to Table 2, plus some background information on sex education in Britain at the time over the span of these data sweeps (Putting the data into context).** “Annual hands-on assessment clinics (known as Focus clinics) were held for all the offspring from the age of 7 years, and this paper describes data collected in 2003/4 at Focus@11; in 2004/5 (at 12.5yrs+); in 2005/6 (at 13.5yrs+); in 2006/8 (at 14.5yrs+) and TeenFocus 4 in 2009/10 (at 17 years of age). Additionally, we report on data collected via self-completion questionnaires administered when the young people were 21+ (in 2012-14) and 23+ years of age (in 2014/15). As dates of birth spanned 18 months, each questionnaire was scheduled to the participant’s birthday as closely as possible (or 6 months thereafter in the case of clinics).”

 It would also be useful to give a sense of when the various variables were collected. Table 1 has 1993 beside the rurality variable and 2014 beside the maternal deprivation variable – does indicate that the data for these variables were derived from different waves of the data?

**Response: Apologies for the ambiguities with the variable naming.  In the case of variable Jan1993aur01ind_m, this is based on 2001 Census data applied to the home address of the mother at the time of the first questionnaire’s completion and return to the study centre (potentially between 1990-1992). For the IMD variable: Jan2014imd2010q5_m is IMD score in quintiles for the 2010 IMD release for timepoint Jan 2014 for mothers, the classification of area being based on the 2010 Census.**  

 For Focus at 11, TF1-TF3 - Given that only the data pertaining to sexual activity with a member of the opposite sex, it would be important to highlight from early on, that the focus is on heterosexual sexual development and activity for these waves of the study. It should be made clear whether it was only these 1548 children that were tracked through for the adolescent years.

**Response: It should be noted that earlier sweeps (those at 11 and 12 years) focussed on heterosexual experiences, but from age 13 the participants were asked if experiences had occurred with either sex.  This is made clear in the tables’ column headers.  This paper only reports actual data collected at each sweep.  Whilst it is possible to analyse these data using only those participants that completed data at each time point, the totals presented in this paper may not always include the same participants.  Any participant may opt out of answering any question, of any data collection wave or from the study altogether.  Conversely, those that have ‘left’ may also rejoin at any time. Table 2 indicates the numbers completing each wave split by sex (where it can be seen that a roughly 50/50 split for each sex completed waves up to 15 years, but thereafter more females than males participated).  In regard to your comment about the 1548 children who admitting kissing in F@11, only those were asked the following question on ‘cuddling’; only those who answered yes to this question were then asked the following one on laying down together, and so on, as stipulated by the ASAI scale (Hansen et al., 1999).  So at age 11.5, about a third (2176) had hugged and for each successive question the number reduced to <5 for all those from ‘laid down together’ onwards.**

 It would be useful to provide the n and inclusion and exclusion criteria for each section. There seems to be a larger n for the adult data.

**Response: Table 2 gives the total numbers completing the clinic session or questionnaire at each time point. There were no exclusions, as all questions are optional to complete.  Missing data would include those who omitted a particular question, section or did not attend the clinic/return questionnaire.  For example, by age 11 about 50% of the original children were still participating. In the demographics table (Table 1), we have used maternal variables collected during the index pregnancy and this also includes the ethnicity of the child (hence the higher n).  The only other exclusion in the published data are higher order births – as these would be potentially identifying. Twins are included.**

 If any celibate participants (and asexual participants for the sexual orientation variables) were included in the analysis.

**Response:** **No analyses as such were carried out for this descriptive paper, only the split by sex assigned at birth.  We only describe the % frequencies (actual numbers are omitted because of small cell counts <5, which may = zero).  Celibate and asexual participants would come under the ‘Other’ category at age 23. A later data sweep (in 2022) has presented many more tick options to cover more LBGTQ+ categories (these are presented in a soon-to be-published data note (Iles-Caven Y, Golding J, Joinson C, Fraser A, Northstone K.  Sexual attitudes, enjoyment and regret in the parent and offspring generations of the Avon Longitudinal Study of Parents and Childhood (ALSPAC): 2022 data sweeps. Wellcome Open Res 2024; 23059).**

 There is a limitation on the generalizability of a predominantly white sample - 95%- which is noted. There has been a significant demographic shift since the data were collected. This could be highlighted when noting the time-period in which the data was collected. **Response: Added in the Methods section:**

**“As with all longitudinal studies, attrition is a problem which is strongly associated with socioeconomic disadvantage. At the time of enrolment there were very few Non-White families resident in the Avon area, which was centred on the city of Bristol (~6%) and therefore research results cannot be extrapolated to cover Non-White populations in general. Over the last 30 years, the numbers of Black, Asian and Minority Ethnic people living in Bristol rose from 5.1% in 1991 to 18.9% in 2021 (Bristol City Council, 2023). The cohort considered in this data note are children born in 1991-2.**

 I would suggest including the limited focus on heterosexual relationships and development in the limitations of the data.

 ​​​​​​​ **Response: Added in Limitations** **“A further limitation is the focus on heterosexual relationships and development, especially within the earlier data collection sweeps”**
